# Cognitive Function and Ophthalmological Diseases: The Beijing Eye Study

**DOI:** 10.1038/s41598-018-23314-5

**Published:** 2018-03-19

**Authors:** Jost B. Jonas, Wen Bin Wei, Li Ping Zhu, Liang Xu, Ya Xing Wang

**Affiliations:** 1 Beijing Institute of Ophthalmology and Beijing Ophthalmology and Visual Science Key Lab, Beijing Tongren Eye Center, Beijing Tongren Hospital, Capital Medical University, Beijing, China; 2 Department of Ophthalmology, Medical Faculty Mannheim of the Ruprecht-Karls-University of Heidelberg, Heidelberg, Germany; 3 Beijing Tongren Eye Center, Beijing Tongren Hospital, Capital Medical University, Beijing, China; 4 Department of Neurology, Beijing Tongren Hospital, Capital Medical University, Beijing, China

## Abstract

To examine associations between cognitive function and ophthalmological parameters, the population-based Beijing Eye Study examined ophthalmologically and physically 3127 individuals (mean age: 64.2 ± 9.8 years). Using the mini–mental state examination, cognitive function was assessed as cognitive function score (CFS). Mean CFS was 26.3 ± 3.7 (median: 27; range: 2–30). Prevalence of mild (CFS: 23–19), moderate (CFS: 18–10) and severe cognitive dysfunction was 9.6% (95% confidence interval (CI): 8.5, 10.6), 3.2% (95% CI: 2.6, 3.9) and 0.6% (95% CI: 0.4,0.9), respectively. In multivariate analysis, better cognition (i.e., higher CFS) was significantly associated with better best corrected visual acuity (r^2^ = 0.38), smaller amount of undercorrected visual acuity, lower prevalence of primary angle-closure glaucoma, and thicker subfoveal choroidal thickness. Prevalence of age-related macular degeneration, open-angle glaucoma, diabetic retinopathy, any type of cataract, retinal vein occlusions or pseudoexfoliation was not significantly correlated with CFS. Though the causal relationship is unclear, the associations of lower cognitive function with undercorrected visual acuity suggest the need for earlier and more regular refraction testing in the elderly so that providing adequate glasses to the elderly can be provided and vision-associated cognitive decline can be reduced. Associations of cognitive function with primary angle-closure glaucoma and leptochoroid should be further explored.

## Introduction

Cognitive impairment is an important cause of morbidity in elderly persons. Its pathogenesis has remained poorly understood so far^1^. Cognitive dysfunction is associated with a multitude of risk factors and disorders. Cerebral microvascular disorders have been discussed to contribute to the development of vascular cognitive impairment and to modify the risk and course of Alzheimer’s disease^2–4^. Except for associations of retinal microvascular abnormalities, early age-related macular degeneration or visual loss with cognitive impairment, correlations between cognitive dysfunction and ocular parameters or ophthalmological diseases have usually not been examined in a systematic manner, yet^5–13^. The knowledge about such associations may be useful to better understand the factors leading to cognitive impairment and vice versa, to better assess how cognitive dysfunction may influence the development and progression of ocular diseases, e.g. by changing the adherence to the recommended therapy. We therefore conducted this study to examine the prevalence of a reduced cognitive function in a populations living in an urban or rural region of Greater Beijing and to asses potential associations between cognitive dysfunction and ocular variables^14–17^.

## Methods

The Beijing Eye Study 2011 is a population-based cross-sectional study which was conducted in a rural region and an urban region of Greater Beijing. According to the Declaration of Helsinki, the Medical Ethics Committee of the Beijing Tongren Hospital approved the study and all participants gave informed written consent. The ethics committee confirmed that all methods were performed in accordance with the relevant guidelines and regulations. Out of 4403 eligible individuals, 3468 subjects (1963 (56.6%) women) participated (response rate: 78.8%). The mean age was 64.6 ± 9.8 years (median 64 years; range 50–93 years). The study population and the study design have been described in detail previously^18,19^.

All examinations were carried out in the communities, either in schoolhouses or in community houses. The study participants underwent a structured interview by trained research technicians. The interview included standardized questions on demographic parameters and on the socioeconomic background, such as the level of education, occupation and family income, questions about the diet, alcohol consumption, smoking and about known diagnosis and current treatment of major systemic diseases like arterial hypertension and hypotension, diabetes mellitus, thyroid disorders, cerebral hemorrhages, coronary heart diseases and hyperlipidemia. The level of education was categorized into the levels of “illiteracy”, “half illiteracy with knowledge of some Chinese words”, “primary school education”, “middle school education”, and “college or higher education”. The ophthalmological examination consisted of automatic refractometry (Auto Refractometer AR-610; Nidek Co., Ltd, Tokyo, Japan), measurement of presenting visual acuity, uncorrected visual acuity and best-corrected visual acuity, tonometry, slit lamp based biomicroscopy of the anterior and posterior segment of the eyes, and photography of the cornea and lens (Neitz CT-R camera; Neitz Instruments Co., Tokyo, Japan), and photography of the macula and optic disc in medical mydriasis (fundus camera; Type CR6–45ΜM; Canon Inc., Tokyo, Japan). Undercorrected visual acuity was defined as the difference between presenting visual acuity and best corrected visual acuity of the better eye. Using the fundus photographs and spectral-domain optical coherence tomographs taken with the enhanced depth imaging modality (Spectralis; Heidelberg Engineering, Heidelberg, Germany), we measured the dimensions of the optic nerve head, parapapillary regions and macula^20^. Applying optical low-coherence reflectometry (Lenstar 900 Optical Biometer; Haag-Streit, Koeniz, Switzerland), we determined the ocular biometric parameters such as the anterior corneal curvature, central corneal thickness, anterior chamber depth, lens thickness and axial length.

The degree of cataract was assessed using the lens photographs. The degree of nuclear opacities was assessed in 6 grades using the grading system of the Age-Related Eye Disease Study. In addition, retro-illuminated photographs of the lens were obtained (Neitz CT-R camera; Neitz Instruments Co., Tokyo, Japan), and the percentage of the areas with cortical and posterior subcapsular lens opacities was measured using a grid. The standard to diagnose a nuclear cataract was a nuclear cataract grade of 4 or more, the standard to diagnose a posterior subcapsular cataract was an amount of posterior subcapsular opacities of 0.01 or more, and the standard to diagnose a cortical cataract was an amount of cortical opacities of 0.05 or more. The degree of fundus tessellation defined as the visibility of the large choroidal vessels was examined on the fundus photographs of the macula and of the optic disc as described in detail previously^19^. It was graded using a scale which ranged from “0” for “no tessellation” to “3” for “marked tessellation”. Diabetic retinopathy was examined on the fundus photographs using the Early Treatment of Diabetic Retinopathy Study (ETDRS) criteria. The minimum criterion for diagnosis of diabetic retinopathy was the presence of at least one microaneurysm. The diagnosis for each individual was based on the grading of the individual´s eye with the highest stage of diabetic retinopathy. Glaucomatous optic neuropathy was defined using the criteria of the International Society of Geographic and Epidemiological Ophthalmology ISGEO. Pseudoexfoliation was assessed by an experienced ophthalmologist during the slit lamp assisted biomicroscopy of the anterior segment after pupillary dilation. The diagnosis of pseudoexfoliation was definite, if the lens surface showed a central whitish coating with a diameter of little less than the normal pupillary diameter, or if the periphery of the lens surface showed a whitish coating which was anteriorly bordered by a darker ring-like region on the lens surface. The assessment of pseudoexfoliation was performed only in phakic eyes. For the diagnosis of age-related macular degeneration, the International ARM (Age-related Maculopathy Epidemiological Study Group) Grading system was used.

Using the mini–mental state examination or Folstein test, we assessed the cognitive function^21,22^. Arterial hypertension was defined as a systolic blood pressure ≥160 mm Hg and/or a diastolic blood pressure ≥95 mm Hg, and/or self-reported current treatment for arterial hypertension with antihypertensive medication. Diabetes mellitus was defined by a fasting blood glucose concentration ≥7.0 mmol/L, an HbA1c value ≥6%, by a self-reported history of physician diagnosis of diabetes mellitus or by a history of drug treatment for diabetes (insulin or oral hypoglycemic agents). The body height was determined in a standardized manner with the shoes routinely removed. The subjects were asked to stand upright as much as possible and with the head raised upright as much as possible. We used a stadiometer as measuring instrument. Depressive symptoms were evaluated using a Chinese depression scale adapted from the Zung Self-Rating Depression Scale^23^. The Chinese depression scale used in our study was validated previously^24,25^. The cerebrospinal fluid pressure was estimated as already described^26^. We applied the formula: CSFP [mmHg] = 0.44 × Body Mass Index [kg/m^2^] + 0.16 × Diastolic Blood Pressure [mmHg] − 0.18 × Age [Years] −1.91. Based on an adaptation of the “Modification of Diet in Renal Disease (MDRD)” equation on the basis of data from Chinese chronic kidney disease patients, the estimated glomerular filtration rate (eGFR) was calculated as “eGFR_MDRD_ = 175 × (Serum Creatinine Concentration(mg/dL))^−1.234^ × Age(Years)^−0.179^ [if female, × 0.79]”, and reduced renal function was defined as an eGFR of less than 60 mL/min per 1·73 m²^27^. Quality of life was assessed by standardized questions on mobility, self-care, performing usual daily activities, presence of pain/discomfort, and on the presence of anxiety/depression (Table 1). The total quality of life score was the sum of the answers given to the 5 questions. A higher score indicated a lower quality of life. Fasting blood samples were collected for measurement of blood lipids, glucose, glycosylated hemoglobin HbA1c and serum creatinine.

**Table 1:** Associations between the cognitive function score and systemic and ocular parameters in the Beijing Eye Study after adjusting for age and level of education.

Parameter	*P*-Value	Standardized Regression Coefficient Beta	Non-Standardized Regression Coefficient B	95% Confidence Interval of B
Gender (Men/Women)	0.90	−0.02	−0.01	−0.23, 0.20
Region of Habitation (Rural/Urban)	<0.001	−0.07	−0.52	−0.78, −0.26
Body Mass Index (kg/m^2^)	0.009	0.04	0.04	0.01, 0.06
Body Height (cm)	0.01	0.04	0.02	0.00, 0.03
Smoking Package Years	0.21	0.02	0.06	−0.03, 0.16
Smoking Ever	0.19	0.02	0.15	−0.07, 0.31
How Many Days Per Week Do You Walk?	0.31	0.02	0.02	−0.02, 0.06
How Many Days Do You Do Vigorous Sport?	0.20	−0.02	−0.06	−0.14, 0.03
How Many Days Do You Do Moderate to Intensive Sport?	<0.001	0.05	0.06	0.03, 0.09
How Many Hours Per Day Do You Sit in Daytime	0.14	−0.02	−0.03	−0.07, 0.01
Depression Score	<0.001	−0.08	−0.05	−0.06, −0.03
Quality of Life, Depression: I am not anxious or depressed/I am moderately anxious or depressed/I am extremely anxious or depressed	0.95	−0.001	−0.01	−0.33, 0.31
Quality of Life, Everyday Work: I am unable to wash or dress myself/I have some problems with performing my usual activities/I am unable to perform my usual activities	<0.001	−0.13	−1.76	−2.15, −1.37
Quality of Life, Mobility: I have no problems in walking about/I have some problems in walking about/I am confined to bed	<0.001	−0.08	−1.05	−1.42, −0.68
Quality of Life, Pain: I have no pain or discomfort/I have moderate pain or discomfort	0.51	0.01	0.07	−0.14, 0.29
Quality of Life, Self-Care: I have I have no problems in washing or dressing myself/I have some problems in washing or dressing myself/I am unable to wash or dress myself	<0.001	−0.11	−1.86	−2.33, −1.40
Quality of Life Total Score	<0.001	−0.07	−0.25	−0.35, −0.15
Systolic Blood Pressure (mmHg)	0.11	0.02	0.00	0.01, 0.90
Diastolic Blood Pressure (mmHg)	0.15	0.02	0.01	0.00, 0.02
Arterial Hypertension	0.65	0.01	0.05	−0.16, 0.26
Estimated Cerebrospinal Fluid Pressure (mmHg)	0.008	0.05	0.05	0.01, 0.09
Blood Glucose Concentration (mmol/L)	0.08	−0.03	−0.07	−0.15, 0.01
HbA1c (%)	0.03	−0.04	−0.14	−0.26, −0.01
Diabetes Mellitus	0.08	−0.03	−0.27	−0.57, 0.03
Blood Concentration of High-Density Lipoproteins (mmol/L)	0.66	0.01	0.07	−0.24, 0.37
Blood Concentration of Low-Density Lipoproteins (mmol/L)	0.14	0.03	0.11	−0.03, 0.24
Blood Concentration of Triglycerides (mmol/L)	0.46	−0.01	−0.04	−0.14, 0.06
Blood Concentration of Cholesterol (mmol/L)	0.27	0.02	0.07	−0.06, 0.19
Best Corrected Visual Acuity (logMAR)	<0.001	−0.16	−3.46	−4.16,–2.76
Best Corrected Visual Acuity<20/40	<0.001	−0.11	−2.10	−2.65, −1.55
Best Corrected Visual Acuity<2/20	<0.001	−0.05	−3.64	−5.64, −1.64
Presenting Visual Acuity (logMAR)	<0.001	−0.11	−1.04	−1.34, −0.74
Undercorrected Visual Acuity (logMAR) (Difference between Presenting Visual Acuity and Best Corrected Visual Acuity)	0.001	−0.05	−0.58	−0.92, −0.25
Intraocular Pressure (mmHg)	0.34	−0.01	−0.02	−0.06, 0.02
Refractive Error (Diopters)	0.02	0.03	0.06	0.01, 0.11
Axial Length (mm)	0.04	−0.03	−0.10	−0.19, −0.003
Anterior Corneal Curvature Radius (mm)	0.12	−0.02	−0.33	−0.74, 0.08
Central Corneal Thickness (µm)	0.32	−0.02	−0.002	−0.005, 0.002
Anterior Chamber Depth (mm)	0.26	−0.02	−0.12	−0.34, 0.09
Lens Thickness (mm)	0.66	0.01	0.08	−0.26, 0.42
Subfoveal Choroidal Thickness (µm)	0.001	0.05	0.002	0.001, 0.003
Retinal Nerve Fiber Layer Thickness (µm)	0.03	0.03	0.009	0.001, 0.017
Neuroretinal Rim Area (mm^2^)	0.55	−0.01	−0.10	−0.41, 0.22
Optic Disc Area (mm^2^)	0.85	−0.004	−0.03	−0.34, 0.28
Parapapillary Alpha Zone (mm^2^)	0.38	0.02	0.10	−0.12, 0.31
Parapapillary Beta/Gamma Zone (mm^2^)	0.04	−0.04	−0.12	−0.23, −0.01
Optic Disc-Fovea Distance (mm)	0.02	−0.04	−0.37	−0.69, −0.05
Optic Disc-Fovea Angle (°)	0.11	0.03	0.02	−0.01, 0.05
Keratoconus (>48 Diopters)	0.91	0.00	−0.07	−1.17, 1.05
Nuclear Cataract	0.42	0.02	0.11	−0.15, 0.36
Cortical Cataract	0.03	−0.04	−0.36	−0.68, −0.03
Subcapsular Cataract	<0.001	−0.06	−0.90	−1.40, −0.40
Cataract Surgery	0.87	0.002	0.04	−0.44, 0.52
Open-Angle Glaucoma	0.74	−0.01	−0.10	–0.71, 0.51
Angle-Closure Glaucoma	<0.001	−0.07	−2.05	−2.94, −1.16
Age-Related Macular Degeneration, Early Stage	0.67	−0.01	−0.08	−0.45, 0.29
Age-Related Macular Degeneration, Intermediate Stage	0.15	0.02	0.20	−0.07, 0.42
Age-Related Macular Degeneration, Late Stage	0.60	−0.01	−0.32	−1.53, 0.89
Diabetic Retinopathy	0.30	−0.02	−0.35	−1.00, 0.30
Retinal Vein Occlusion, Total	0.55	−0.01	−0.26	−1.10, 0.58
Central Retinal Vein Occlusion	0.70	0.01	0.49	−2.02, 3.01
Branch Retinal Vein Occlusion	0.44	−0.01	−0.35	−1.24, 0.54
Central Serous Choroidopathy	0.97	0.00	0.00	−0.08, 0.08
Fundus Tessellation	<0.001	−0.06	−0.16	−0.25, −0.08
Prevalence of Pseudoexfoliation	0.68	−0.01	−0.10	−0.56, 0.36

Statistical analysis was performed using a commercially available statistical software package (SPSS for Windows, version 22.0, IBM-SPSS, Chicago, IL, USA). Only the right eye of each study participant was assessed. In a first step, we assessed the mean values of the main outcome parameters, i.e. CFS and ocular and systemic variables, presented as mean ± standard deviation. The mean frequency of a reduced CFS was expressed as mean and 95% confidence interval (CI). In a second step, we performed a linear regression analysis with the CFS as dependent variable. Since in this univariate analysis a higher CFS was strongly dependent on younger age and higher level of education, we tested associations between the CFS and any other parameter after adjusting for age and level of education. In a third step of the analysis, we included into a multivariate regression analysis all those parameters which were associated with the CFS after adjusting for age and the educational level. We then dropped step by step those parameters for which either the collinearity was too high or which no longer were significantly associated with the CFS. For this multivariate analysis, we did not correct the *P-*values for performing multiple comparisons as it would have been necessary in a univariate analyses, since a multivariate approach takes into account the potential associations between the independent variables. In a fourth step, we performed a binary regression analysis with the presence of a reduced CFS, defined as a CFS ≤ 24 (or the presence of mild, moderate or severe cognitive dysfunction) as dependent variable and the ocular and other general parameters as independent variables. We calculated the standardized regression coefficient beta and the non-standardized regression coefficient B and its 95% CI. In the binary regression analysis, we determined the odds ratio (OR) and its 95% CI. All *P*-values were two-sided and considered statistically significant, if the values were less than 0.05.

The datasets generated or analyzed during the current study are promptly available on request from the corresponding author without undue qualifications in material transfer agreements.

## Results

The study included 3127 individuals (1359 (43.5%) men) for whom measurements of cognitive function were available. The mean age was 64.2 ± 9.8 years (median: 63 years; range: 50–93 years), the average axial length was 23.2 ± 1.1 mm (median: 23.1 mm; range: 13.39–30.88 mm) and the mean refractive error was −0.19 ± 2.07 diopters (median: +0.25 diopters; range: −22.0 to +7.00 diopters) (Table 2). The group of individuals included into the study and the group of individuals not participating did not differ significantly in gender (1359/1768 versus 146/195; *P* = 0.86), prevalence of arterial hypertension (44.1% (95%: 42.4, 45.9) versus 40.8% (95%: 35.4, 46.2); *P* = 0.27), prevalence of diabetes mellitus (19.5% (95% CI: 18.0, 21.1) versus 27.1% (19.7, 34.6); *P* = 0.10), and ocular axial length (23.2 ± 1.1 mm versus 23.4 ± 1.5 mm; *P* = 0.24). Both groups differed significantly in age (64.2 ± 9.8 years versus 68.6 ± 9.4 years; *P* < 0.001) and in refractive error (−0.19 ± 2.07 diopters versus −1.79 ± 2.50 diopters; *P* = 0.003).

**Table 2:** Demographic data and general and ocular parameters of the study population and prevalence of systemic and ocular diseases.

Parameter	Mean ± Standard Deviation (Median)/Prevalence (95% Confidence Interval (CI))
Systemic Parameters	
Age (Years)	64.2 ± 9.8 (63)
Body Mass Index (kg/m^2^)	25.6 ± 3.9 (25.2)
Body Height (cm)	161.8 ± 8.2 (161.5)
Smoking Package Years	9.7 ± 18.7 (0)
Smoking Ever	31.7% (95% CI: 30.0, 33.3)
Systolic Blood Pressure (mmHg)	130.4 ± 20.6 (129)
Diastolic Blood Pressure (mmHg)	70.0 ± 12.4 (69)
Mean Blood Pressure (mmHg)	90.1 ± 14.0 (89)
Arterial Hypertension	53.2% (95% CI: 51.5, 55.0)
Diabetes mellitus	19.5% (18.0, 21.1)
Blood Concentration (mmol/L) of	
Glucose	5.72 ± 1.56 (5.34)
High-Density Lipoproteins	1.44 ± 0.39 (1.39)
Low-Density Lipoproteins	3.37 ± 0.87 (3.33)
Triglycerides	1.62 ± 1.18 (1.31)
Cholesterol	5.06 ± 0.96 (5.03)
Creatinine	66.9 ± 16.1 (64.4)
C-Reactive Protein	1.95 ± 4.09 (0.96)
Ophthalmological Parameters	
Best Corrected Visual Acuity (logMAR)	0,05 ± 0.16 (0.00)
Presenting Visual Acuity (logMAR)	0.27 ± 0.37 (0.27)
Undercorrected Visual Acuity (logMAR) (Difference between Presenting Visual Acuity and Best Corrected Visual Acuity)	0.22 ± 0.31 (0.18)
Intraocular Pressure (mmHg)	14.5 ± 2.7 (14.0)
Refractive Error (Diopters)	−0.19 ± 2.07 (0.25)
Axial Length (mm)	23.2 ± 1.1 (23.1)
Anterior Corneal Curvature Radius (mm)	7.62 ± 0.25 (7.62)
Central Corneal Thickness (µm)	532 ± 33 (532)
Anterior Chamber Depth (mm)	2.49 ± 0.49 (2.44)
Lens Thickness (mm)	4.56 ± 0.33 (4.56)
Subfoveal Choroidal Thickness (µm)	256 ± 108 (252)
Retinal Nerve Fiber Layer Thickness (µm)	101 ± 12 (102)
Keratoconus (>48 Diopters)	0.9% (95% CI: 0.8, 1.2)
Nuclear Cataract	43.1% (95% CI: 41.1, 45.0)
Cortical Cataract	17.0% (95% CI: 15.5, 18.5)
Subcapsular Cataract	5.9% (95% CI: 4.9, 6.8)
Cataract Surgery	0.5% (95% CI: 0.3, 0.8)
Open-Angle Glaucoma	2.9% (95% CI: 2.3, 3.5)
Angle-Closure Glaucoma	1.3% (95% CI: 0.9, 1.7)
Age-Related Macular Degeneration, Early Stage	8.2% (95% CI: 7.0, 9.0)
Age-Related Macular Degeneration, Intermediate Stage	17.5% (95% CI: 16.0, 19.0)
Age-Related Macular Degeneration, Late Stage	0.7% (95% CI: 0.2, 0.10)
Diabetic Retinopathy	2.6% (95% CI: 2.0, 3.1)
Retinal Vein Occlusion, Total	2.9% (95% CI: 2.3, 3.5)
Central Serous Choroidopathy	0.3% (95% CI: 0.0, 1.0)
Fundus Tessellation	1.64 ± 1.30 (2.00)
Prevalence of Pseudoexfoliation	6.3% (95% CI: 5.4, 7.2)

**Figure 1:**
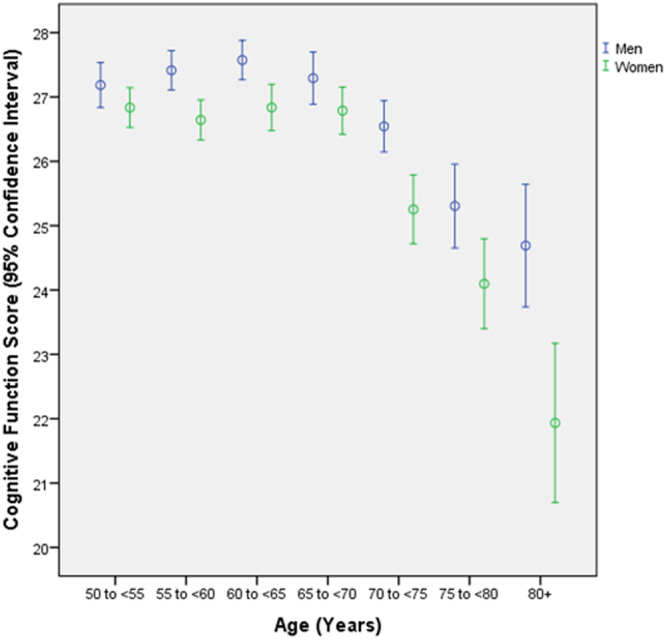
Distribution of the cognitive function score in the Beijing Eye Study, stratified by age.

The mean CFS was 26.3 ± 3.7 (median: 27; range: 2–30) (Fig. 1). The CFS increased with age, ranging from a median of 28 for those 55 to 69 years of age, to 26 for individuals 70 to 79 years of age, and to 25 for individuals aged 80+ years. The median CFS was 28 for individuals with middle school as level of education or with high school or higher level of education, and 25 for those with primary school as level of education. These values were comparable with normative CSF values found in previous studies such as the study by Crum and colleagues, in which the median CSF was 29 for individuals 18 to 24 years of age and 25 for individuals 80 years of age and older, and in which the median CSF 29 for individuals with at least 9 years of schooling, 26 for those with 5 to 8 years of schooling, and 22 for those with 0 to 4 years of schooling^22^. Similar normative value were reported in other studies^28,29^.

**Figure 2:**
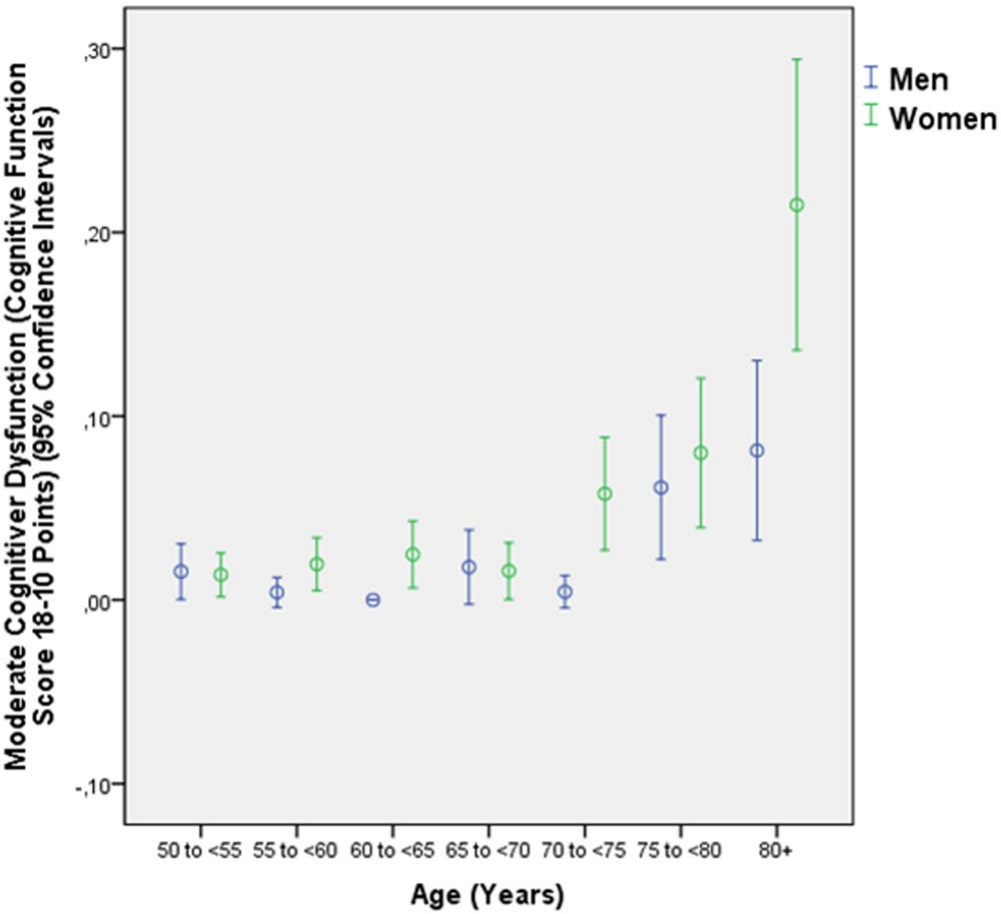
Distribution of the frequency moderate cognitive dysfunction, defined as a cognitive function score ranging between 18 and 10 points, in the Beijing Eye Study, stratified by age.

**Figure 3:**
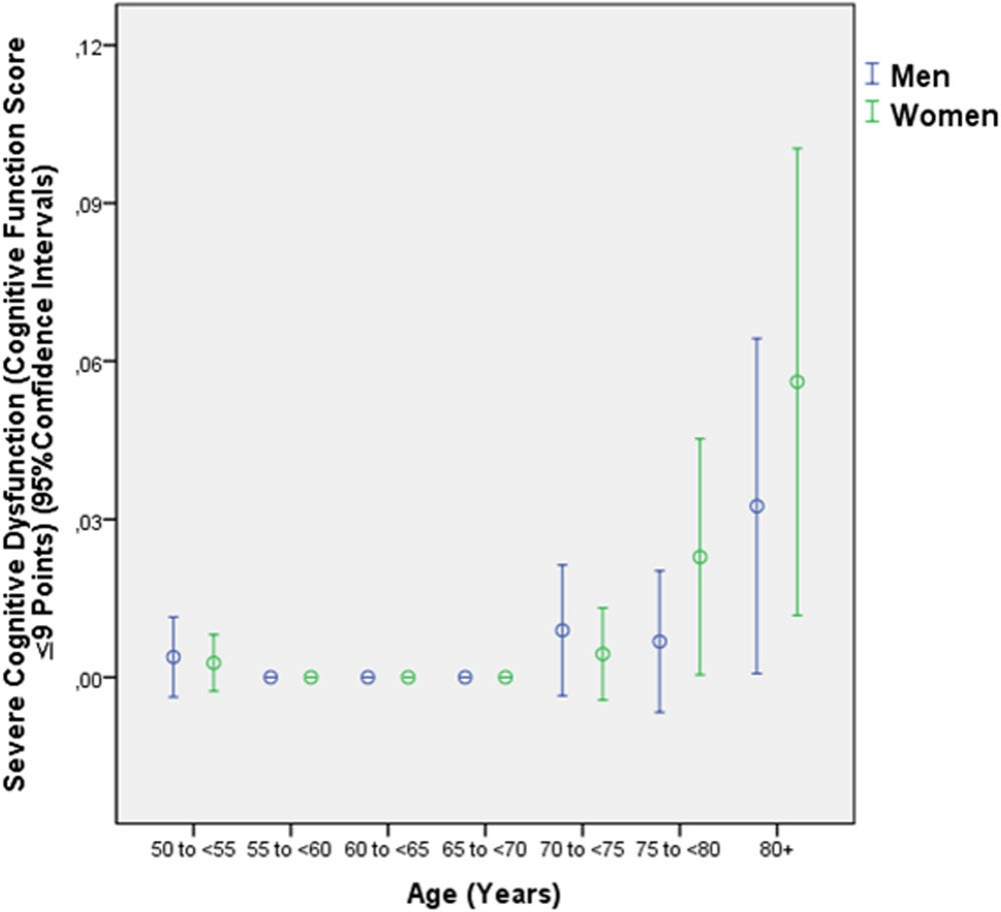
Distribution of the frequency severe cognitive dysfunction, defined as a cognitive function score <10 points, in the Beijing Eye Study, stratified by age.

In the subgroup of individuals with presenting visual acuity of 20/20 or better, the mean CFS was 27.3 ± 2.5 (median: 28; range: 8–30). Out of the 3127 study participants, 420 (13.4% (95% CI: 12.2, 14.6)) individuals had a CFS of ≤ 23. Defined as a CFS ranging between 23 and 19 points, mild cognitive dysfunction was present in 299 individuals (9.6%; 95% CI: 8.5, 10.6). Defined as a CFS ranging between 18 and 10 points, moderate cognitive dysfunction was present in 101 individuals (3.2%; 95% CI: 2.6, 3.9) (Fig. 2). Defined as a CFS of ≤ 9 points, severe cognitive dysfunction was present in 20 individuals (0.6%; 95% CI: 0.4, 0.9) (Fig. 3).

In univariate analysis, better cognition (i.e. higher CFS) was associated with younger age (*P* < 0.001; correlation coefficient r: −0.27) and higher level of education (*P* < 0.001; correlation coefficient r: 0.57). A similar result was obtained if both, age (*P* < 0.001; beta: −0.24; B: −0.09; 95% CI: −0.10, −0.08) and educational level (*P* < 0.001; beta: 0.56; B: 1.92; 95% CI: 1.82, 2.02) were added as independent variables into a multivariate analysis with CFS as dependent variable. In view of these statistically strong associations, all further parameters were tested for an association with CFS after adjusting for age and level of education. It revealed associations with several systemic and ocular parameters (Table 1).

The multivariate analysis included the CFS score as dependent variable and as independent variables all those parameters which had a *P*-value < 0.10 for their univariate association with the CFS score. After dropping parameters due to collinearity or due to missing significance in the final model, a higher cognitive score was significantly (regression coefficient r^2^ = 0.38) associated with younger age (*P* < 0.001), higher level of education (*P* < 0.001), rural region of habitation (*P* < 0.001), lower depression score (*P* < 0.001), higher physical activity (“How many days do you do moderate to intensive sports or similar activities?”) (*P* = 0.03), better presenting visual acuity (*P* = 0.02), better best corrected visual acuity (*P* < 0.001), lower prevalence of primary angle-closure glaucoma (*P* < 0.001), and lower degree of fundus tessellation (*P* = 0.002) (Table 3). If a Bonferroni correction was performed for correcting for performing of multiple comparisons, the associations of most of the parameters (except for physical activity and presenting visual acuity) would have remained statistically significant (Table 3). If gender was added to the model, it was not significantly (*P* = 0.10; beta: 0.03) correlated with cognitive function. The prevalence of age-related macular degeneration (any type) (*P* = 0.92), open-angle glaucoma (*P* = 0.80), diabetic retinopathy (*P* = 0.99), nuclear cataract (*P* = 0.40), cortical cataract (*P* = 0.27), posterior subcapsular cataract (*P* = 0.50), retinal vein occlusions (*P* = 0.76) or pseudoexfoliation (*P* = 0.96), if added as single parameters to the model, were not significantly associated with the CFS. Replacing fundus tessellation by subfoveal choroidal thickness revealed a significant positive association (*P* = 0.005; beta: 0.05; B: 0.002; 95% CI: 0.000, 0.003) of the latter with the CFS. If presenting visual acuity was replaced by undercorrected visual acuity, a smaller amount of undercorrected visual acuity was significantly associated with a higher CFS (*P* = 0.02; beta: 0.04; B: 0.40; 95% CI: 0.74, 0.06).

**Table 3:** Associations (multivariate analysis) between the cognitive function score and systemic and ocular parameters in the Beijing Eye Study.

Parameter	*P*-Value	Standardized Regression Coefficient Beta	Non-Standardized Regression Coefficient B	95% Confidence Interval of B	Variance Inflation Factor
Age (Years)	<0.001	−0.10	−0.04	−0.05, −0.02	1.68
Level of Education	<0.001	0.55	1.85	1.73, 1.97	1.51
Region of Habitation (Rural/Urban)	<0.001	−0.08	−0.53	−0.78, −0.27	1.65
Depression Score	<0.001	−0.08	−0.05	−0.07, −0.03	1.04
Physical Activity (“How Many Days Do You Do Moderate-Intensive Sports or Similar Activities?”)	0.03	0.03	0.04	0.01, 0.07	1.11
Presenting Visual Acuity (logMAR)	0.02	−0.04	−0.40	−0.74, −0.06	1.42
Best Corrected Visual Acuity (logMAR)	<0.001	−0.10	−2.34	−3.27, −1.41	1.69
Primary Angle-Closure Glaucoma	<0.001	−0.07	−1.94	−2.82, −1.07	1.03
Fundus Tessellation	0.002	−0.05	−0.13	−0.22, −0.05	1.25

If in a reverse manner the presence of primary angle-closure glaucoma was the dependent variable in a binary regression analysis, higher prevalence of primary angle-closure glaucoma was associated with older age (*P* < 0.001; OR: 1.07; 95% CI: 1.04, 1.11) and worse cognition (*P* = 0.002; OR: 0.92; 95% CI: 0.87, 0.97) and with a tendency of a lower body height (*P* = 0.15; OR: 0.97; 95% CI:0.94, 1.01).

If the whole study population was divided into a subgroup with a CFS ≤ 24 and another subgroup including the remaining participants, the subgroup with the lower CFS was significantly correlated with older age (*P* < 0.001; OR: 1.04; 95% CI: 1.03, 1.06), lower level of education (*P* < 0.001; OR: 0.28; 95% CI: 0.24, 0.28), worse best corrected visual acuity (*P* < 0.001; OR: 8.49; 95% CI: 3.34, 21.6) and higher degree of fundus tessellation (*P* = 0.03; OR: 1.11; 95% CI: 1.01, 1.21), while physical activity (*P* = 81), region of habitation (*P* = 0.51), prevalence of primary angle-closure glaucoma (*P* = 0.12) and the depression score (*P* = 0.053) were not significantly associated with the prevalence of a CSF ≤ 24. The prevalence of age-related macular degeneration (any type) (*P* = 0.57), open-angle glaucoma (*P* = 0.80), diabetic retinopathy (*P* = 0.60), nuclear cataract (*P* = 0.42), cortical cataract (*P* = 0.42), posterior subcapsular cataract (*P* = 0.21), retinal vein occlusions (*P* = 0.60) or pseudoexfoliation (*P* = 0.28), if added as single parameters to the model, were not significantly associated with the prevalence of a low CFS. If best corrected visual acuity was replaced by undercorrected visual acuity, a higher amount of undercorrected visual acuity was significantly associated with higher prevalence of the decreased cognitive dysfunction (*P* = 0.04; OR: 0.72; 95% CI: 0.52, 0.99).

A higher prevalence of mild cognitive dysfunction (defined as a CFS ranging between 23 and 19 points) was correlated with older age (*P* = 0.02; OR: 1.02; 95% CI:1.00, 1.05), lower level of education (*P* < 0.001; OR: 0.17; 95% CI: 0.15, 0.21), higher degree of fundus tessellation (*P* < 0.001; OR: 1.28; 95% CI: 1.12, 1.46), higher prevalence of primary angle-closure glaucoma (*P* = 0.01; OR: 3.16; 95% CI: 1.03, 9.65), and marginally significantly with a lower best corrected visual acuity (*P* = 0.06; OR: 2.64; 95% CI: 0.97, 7.21), while the depression score (*P* = 0.66), gender (*P* = 0.58), physical activity (*P* = 0.59) and region of habitation (*P* = 0.21) were not significantly associated with the prevalence of mild cognitive dysfunction.

Higher prevalence of moderate cognitive dysfunction was correlated with older age (*P* < 0.001; OR: 1.07; 95% CI:1.05, 1.10), lower level of education (*P* < 0.001; OR: 0.31; 95% CI: 0.25, 0.37), higher depression score (*P* < 0.001; OR: 1.07; 95% CI: 1.04, 1.10) and lower physical activity (“How many days do you do moderate to intensive sports or similar activities?”) (*P* = 0.03; OR: 0.90; 95% CI: 0.83, 0.99), while best corrected visual acuity (*P* = 0.36), region of habitation (*P* = 0.29), fundus tessellation (*P* = 0.10) and the prevalence of angle-closure glaucoma (*P* = 0.08) were not significantly associated with the prevalence of moderate cognitive dysfunction.

Higher prevalence of severe cognitive dysfunction was correlated with older age (*P* < 0.001; OR: 1.11; 95% CI:1.05, 1.18), lower level of education (*P* < 0.001; OR: 0.40; 95% CI: 0.28, 0.57) and marginally with lower best corrected visual acuity (*P* = 0.056; OR: 3.03; 95% CI: 0.97, 9.49), while region of habitation (*P* = 0.89), fundus tessellation (*P* = 0.79), physical activity (“How many days do you do moderate to intensive sports or similar activities?”) (*P* = 0.61), prevalence of angle-closure glaucoma (*P* = 0.34), depression score (*P* = 0.16) were not significantly associated with the prevalence of severe cognitive dysfunction.

## Discussion

Our population-based study performed in a rural area and an urban region of Greater Beijing revealed a prevalence of mild cognitive dysfunction of 9.6%, of moderate cognitive dysfunction of 3.2%, and of severe cognitive dysfunction of 0.6% (Figs 2, 3). Higher cognitive function correlated with younger age, higher level of education, rural region of habitation, lower depression score, higher physical activity, better best corrected visual acuity, smaller amount of undercorrected visual acuity, lower prevalence of primary angle-closure glaucoma and lower degree of fundus tessellation (or with thicker subfoveal choroidal thickness as a surrogate). The prevalences of age-related macular degeneration, open-angle glaucoma, diabetic retinopathy, nuclear cataract, cortical cataract, posterior subcapsular cataract, retinal vein occlusions or pseudoexfoliation were not significantly associated with cognitive function.

Some of associations between higher cognitive function and higher educational level and younger age as described in our study have been reported in numerous previous studies^30–33^. Lower cognitive function was associated with the ocular parameters of lower best corrected visual acuity, higher amount of undercorrected visual acuity, lower prevalence of primary angle-closure glaucoma and lower degree of fundus tessellation, while the prevalence of major ocular diseases including age-related macular degeneration, open-angle glaucoma, diabetic retinopathy, nuclear cataract, cortical cataract, posterior subcapsular cataract, retinal vein occlusions or pseudoexfoliation were not significantly associated with cognition (i.e. the CFS). These findings suggested that the major ocular factors associated with cognitive dysfunction were a reduced best corrected visual acuity and a higher amount of undercorrected visual acuity, independently of the cause of reduced vision, except for primary angle-closure glaucoma. It might indicate that patients with major ocular diseases, after adjusting for visual acuity, have a normal cognitive function in relationship to other systemic factors such as their age and level of education. One may infer that the major ocular diseases, after adjusting for visual acuity, were not a risk for the development of cognitive function. A correlation between decreased vision and reduced cognitive function has also been demonstrated reported in previous investigations^34–37^. Rogers and Langa performed a longitudinal study with a follow-up of 8.5 years on 625 elderly people with normal cognition at baseline and detected that decreased visual acuity was correlated with the incidence of dementia and that subjects with good visual acuity at the start of the study had a 63% decreased probability of dementia as compared to individuals with lower visual acuity^34^. Participants of the Singapore Malay Eye Study with visual impairment had a significantly higher probability to be cognitively disabled^35^.

Besides a low best corrected visual acuity, a higher amount of undercorrected visual acuity was related with a lower cognitive function. This correlation might have been caused by a relative self-neglect of individuals with decreased cognitive function so that they did not notice or did not complain about unsharp vision. Individuals with reduced cognitive function might also no longer have the financial possibilities to obtain glasses correcting their refractive errors. In addition to the undercorrection of refractive error as sequel of a decreased cognitive function, the unsharp vision might also have caused or might further have aggravated a cognitive dysfunction. If one assumes that that visual deprivation as part of sensory deprivation in the elderly may (further) decrease cognitive function, the association between the undercorrection of refractive error and worse cognition in our study population suggests that in particular elderly individuals should be checked for the accuracy of the correction of their refractive errors, especially for near vision^38,39^. Future studies may assess whether providing best correcting glasses to elderly individuals has a mitigating effect on the course of a disease associated with cognitive dysfunction. According to a recent meta-analysis the undercorrection of refractive error was not typical for China but it is a universal problem. Undercorrected refractive error is in all world regions the most common cause for moderate to severe vision impairment, and in some regions even for blindness^40^.

Interestingly, worse cognition was correlated with a higher degree of fundus tessellation. If fundus tessellation was replaced by subfoveal choroidal thickness, a thinner choroidal thickness was associated with worse cognition in the multivariate analysis. It suggests that a leptochoroid, which is a surrogate of a marked fundus tessellation, may be an indicator of a decreased cognitive function. One may assume that the common origin of the blood circulation of the brain and choroid through the internal carotid artery could play a role for the association between a leptochoroid and cognitive dysfunction. Since the choroidal thickness depends on the choroidal blood vessel filling, one may discuss that a reduced cerebral blood circulation might have an effect on both a decreased cognitive function and a reduced choroidal thickness.

Worse cognition was also correlated with a higher prevalence of primary angle-closure glaucoma in the multivariate analysis of our study. In a reverse manner, a higher presence of primary angle-closure glaucoma was associated with older age (*P* < 0.001) and worse cognition (*P* = 0.002), and marginally significantly with a lower body height (*P* = 0.15; OR: 0.97; 95% CI:0.94, 1.01). The reasons for the correlation between lower a cognitive function and primary angle-closure glaucoma have remained elusive yet. Previous studies revealed that glaucomatous optic neuropathy was associated with structural brain abnormalities with a decrease in the bilateral gray-matter volume in the visual cortex and other centers such as the lingual gyrus, postcentral gyrus and superior frontal gyrus, and an increase in the bilateral gray matter volume in other centers such as the middle temporal gyrus and inferior parietal gyrus^41,42^. It might hover have been unlikely that these cerebral changes were mainly responsible for the association between angle-closure glaucoma and worse cognition, since the cerebral changes were described in patients with open-angle glaucoma the prevalence of which was not associated with cognition in our study population. Other studies suggested that patients with angle-closure glaucoma had a shorter body height than individuals without angle-closure glaucoma^43^. Correspondingly, a shallow anterior chamber and a narrow chamber angle (both risk factors for the development of primary angle-closure glaucoma) were associated with shorter short body stature after adjusting for older age, female gender, hyperopia, nuclear cataract and other factors^44^. Lower body height is associated with a lower socioeconomic background, which correlates with a lower cognitive function score^45^. Although this sequence of associations may explain the correlation between a higher prevalence of angle-closure glaucoma and worse cognition, it has to be taken into account, that the present study was not focused on primary angle-closure glaucoma and that factors potentially confounding the association between primary angle-closure glaucoma and cognitive function might thus not have been included into the statistical analysis.

The prevalence of pseudoexfoliation was not correlated with worse cognition after adjusting for age and other systemic parameters. It was discussed in a former investigation that pseudoexfoliation might be correlated with Alzheimer’s disease, since both disorders share similarities with respect to the detection of amyloid-associated proteins^46,47^. In our investigation, the CFS was not significantly associated with the occurrence of pseudoexfoliation, so that the findings of our study do not support the notion of an association between cognitive dysfunction and pseudoexfoliation.

Finally, we did not find a significant association between the retinal nerve fiber layer thickness and cognitive function in the multivariate analysis. In the European Prospective Investigation of Cancer (EPIC) Norfolk cohort study, thicker measurements of the retinal nerve fiber layer thickness were associated with a higher cognitive function score after adjusting for age, gender, educational level, visual acuity, axial length and other parameters^48^. The authors however concluded however that the associations were weak and were not currently of predictive value.

When the results of our study are discussed, the study´s limitations should be considered. First, a major disadvantage of the utilization of the mini–mental state examination was that it was affected by demographic factors, mostly by age and educational level. Both parameters were however, included into the multivariate analysis in the present study. Second, another disadvantage of the mini–mental state examination was that it might have lacked sensitivity to mild cognitive impairment and it might have failed to adequately discriminate patients with mild Alzheimer’s disease from normal patients. In the present study, however, we assessed correlations between cognitive function and other parameters not only in a binary regression analysis with the presence of a reduced CFS as dependent variable, but we also assessed associations between the CFS and parameters in a linear manner without having defined cut-off values. Third, we did not assess retinal microvascular abnormalities which were associated with decreased cognitive function in previous investigations^3–6^.

In conclusion, this Northern Chinese population with its prevalence of mild, moderate and severe cognitive dysfunction (9.6%, 3.2% and 0.6%, resp.) was comparable with other populations. Lower cognitive function correlated with lower best corrected visual acuity, higher amount of undercorrected visual acuity, higher prevalence of primary angle-closure glaucoma and higher degree of fundus tessellation (or a leptochoroid as surrogate) after adjusting for older age, higher level of education, rural region of habitation, lower depression score, and higher physical activity. The prevalences of major ocular diseases such as age-related macular degeneration, open-angle glaucoma, diabetic retinopathy, nuclear cataract, cortical cataract, posterior subcapsular cataract, retinal vein occlusions or pseudoexfoliation were not significantly associated with cognitive function. Since lower cognitive function was association with higher amount of undercorrected visual acuity, providing adequate glasses for full correction of refractive errors, including for near vision, may potentially be helpful to reduce cognitive dysfunction. The associations of lower cognitive function with primary angle-closure glaucoma or with a leptochoroid may be further explored.
